# Postauricular gentamicin administration does not provide preferential inner-ear delivery in guinea pigs

**DOI:** 10.7717/peerj.21796

**Published:** 2026-10-27

**Authors:** Dan Chen, Liling Li, Jia Luo, Jingqian Tan, Xuexin Lin, Dalian Ding, Peng Li

**Affiliations:** 1 Department of Otorhinolaryngology, The Third Affiliated Hospital of Sun Yat-Sen University, Guangzhou, Guangdong, China; 2 Department of Otolaryngology Head and Neck Surgery, Zhuhai People’s Hospital, Zhuhai, Guangdong, China; 3 Center for Hearing and Deafness, Department of Communicative Disorders and Sciences, University at Buffalo, Amherst, New York, United States

**Keywords:** Postauricular injection, Blood–labyrinth barrier, Gentamicin, Inner ear drug delivery, Perilymph

## Abstract

**Background:**

Whether postauricular injection provides preferential access to the inner ear relative to conventional systemic administration remains uncertain. We used gentamicin as an ototoxic model drug to compare postauricular, intramuscular and intratympanic injection through a surgically created posterior tympanotomy in guinea pigs.

**Methods:**

Forty-eight male guinea pigs were studied in repeated-dose and single-dose experiments. In the repeated-dose experiment, gentamicin was administered postauricularly or intramuscularly every 12 h for 7 days (14 injections). Auditory brainstem response (ABR) thresholds, cochlear outer hair cells (OHC), body weight, serum creatinine, and blood urea nitrogen were assessed. In the single-dose experiment, animals received gentamicin by intratympanic injection through a posterior tympanotomy, postauricular injection, or intramuscular injection. OHC morphology and perilymph gentamicin concentrations were evaluated at 2.5 and 12.5 h.

**Results:**

Repeated postauricular and intramuscular administration produced comparable bilateral ABR threshold elevations and extensive OHC loss, together with body-weight reduction and renal dysfunction; no significant between-route differences were detected for these outcomes. At 12.5 h after a single administration, moderate OHC loss was observed only after intratympanic injection. Perilymph gentamicin concentrations at 2.5 h were 4,103 ± 349 μg/mL after intratympanic injection, 18.43 ± 4.70 μg/mL after postauricular administration, and 21.84 ± 16.75 μg/mL after intramuscular administration. At 12.5 h, the respective concentrations were 416.20 ± 109.10, 10.86 ± 5.98, and 11.58 ± 7.88 μg/mL. Postauricular and intramuscular concentrations did not differ significantly at either time point.

**Conclusions:**

Postauricular gentamicin injection did not provide preferential access to the inner ear compared with intramuscular injection. Under the conditions of this guinea pig model, its cochlear and systemic effects were consistent with systemic exposure rather than targeted local delivery that bypassed the blood–labyrinth barrier (BLB).

## Introduction

Hearing impairment is one of the most prevalent sensory disorders worldwide. According to the World Health Organization, approximately 1.5 billion people experience some degree of hearing loss, including approximately 430 million who require rehabilitation services (Chadha, Kamenov & Cieza, 2021). Despite recent advances in gene therapy and pharmacological treatment (Lv et al., 2024; Schilder et al., 2024), effective delivery of therapeutic agents to the inner ear remains challenging because of the blood–labyrinth barrier (BLB).

The BLB is a selective vascular interface that separates the systemic circulation from the fluids of the inner ear, thereby maintaining inner-ear homeostasis and restricting the entry of many circulating substances. In the cochlear stria vascularis, the principal structural components of the BLB include capillary endothelial cells interconnected by tight junctions, the associated basement membrane, pericytes, and perivascular resident macrophage-like melanocytes (Ding et al., 2016; Jiang et al., 2020; Yi et al., 2025). The BLB shares certain structural and functional characteristics with the blood–brain barrier (BBB). Therefore, a drug-delivery approach proposed to bypass the BLB should demonstrate preferential inner-ear exposure compared with a conventional systemic route (Du et al., 2026; Saint-Pol et al., 2020).

Systemic administration, including oral, intravenous (IV), and intramuscular (IM) administration, is commonly used to treat sensorineural hearing loss (SNHL). Oral or IV glucocorticoids are the first-line treatments for conditions like sudden hearing loss and Meniere’s disease (Basura et al., 2020; Chandrasekhar et al., 2019). However, systemic treatment may produce limited drug exposure in the inner ear and may also cause systemic adverse effects (Huang et al., 2021; Plontke et al., 2024). Intratympanic administration is an established and generally well-tolerated local approach that places drug in the middle ear for passage mainly across the round-window membrane (Devantier et al., 2022). Its procedural risks are relatively low but include transient dizziness or vertigo, discomfort, otitis media, and tympanic-membrane perforation. In a retrospective series of 278 intratympanic injections, transient dizziness and post-injection vertigo occurred after 16.9% and 1.8% of injections, respectively (Liu et al., 2016). In addition, a randomized trial reported ear infection in 5.4% of patients receiving intratympanic corticosteroid injections (Rauch et al., 2011). A systematic review reported tympanic-membrane perforation rates of approximately 1.0% after direct tympanocentesis and 7.3% when a ventilation tube was used for repeated administration (Kim et al., 2022).

Recent studies have explored the potential of postauricular injection for treating SNHL, suggesting that this method may facilitate drug entry into the inner ear (Lv et al., 2022; Ren et al., 2021). Proposed routes from the postauricular region to the inner ear include absorption into local capillary or lymphatic networks, distribution through venous connections involving the mastoid emissary system, and local spread along soft-tissue, perivascular, or temporal-bone pathways (Chen et al., 2022; Huang, Zhao & Wang, 2020). However, the efficacy of postauricular injections in delivering drugs to the inner ear remains undetermined. To investigate this, we conducted an experiment involving the subcutaneous injection of gentamicin, a drug known to affect sensory hair cells in the inner ear, into the postauricular region of guinea pigs. This study aimed to assess whether postauricular injection could effectively deliver gentamicin into the inner ear, serving as a viable local treatment method.

## Materials and Methods

### Animals and experimental design

Forty-eight healthy male albino guinea pigs, 3 months of age and weighing 280–320 g, were obtained from Guangzhou Longgui Xingke Animal Breeding Farm (Guangzhou, China). Animals were acclimatized for 7 days and housed under a 12-h light/dark cycle at 20 ± 4 °C and 60 ± 5% relative humidity, with free access to food and water. Social housing, hiding devices, and species-appropriate enrichment were provided. All procedures were approved by the Experimental Animal Ethics Committee of Guangdong Province Huawei Testing Co., Ltd. (ethics no. 202208007) and were conducted in accordance with applicable laboratory-animal care guidelines.

The study comprised a repeated-dose experiment and a single-dose experiment. The repeated-dose experiment included an untreated control group, a postauricular group, and an intramuscular group. Auditory brainstem response (ABR), body weight, renal function, and cochlear OHC morphology were evaluated in this cohort. The single-dose experiment included intratympanic injection through a surgically created posterior tympanotomy, postauricular, and intramuscular administration groups assessed at 2.5 and 12.5 h. Cochlear outer hair cells (OHC) morphology and perilymph gentamicin concentrations were assessed in the single-dose route/time-point groups. Group allocation, dosing, terminal time points, and outcomes are summarized in Table 1.

**Table 1:** Experimental groups, dosing regimens, time points, and outcome assessments.

Experiment	Group	*N*	Endpoint	Outcomes
Repeated dose	Untreated control	4	Matched follow-up	ABR; body weight; BUN/Scr; OHC
Repeated dose	Postauricular	4	1 and 7 d after final dose	ABR; body weight; BUN/Scr; OHC
Repeated dose	Intramuscular	4	1 and 7 d after final dose	ABR; body weight; BUN/Scr; OHC
Single dose	Intratympanic	6	2.5 h	Perilymph gentamicin; OHC
Single dose	Postauricular	6	2.5 h	Perilymph gentamicin; OHC
Single dose	Intramuscular	6	2.5 h	Perilymph gentamicin; OHC
Single dose	Intratympanic	6	12.5 h	Perilymph gentamicin; OHC
Single dose	Postauricular	6	12.5 h	Perilymph gentamicin; OHC
Single dose	Intramuscular	6	12.5 h	Perilymph gentamicin; OHC

This was an exploratory animal study. No *a priori* sample-size calculation was performed; the sample size was based on the experimental design, prior laboratory experience, and pilot observations. No animals or data points were excluded from the final analyses. The animal was the experimental unit; when both ears were analyzed, ear was treated as a within-animal factor. ABR measurements were performed by an experimenter blinded to group allocation, and hair-cell counting was conducted independently by observers blinded to the experimental conditions.

Animals were observed at least twice daily, and more frequently on procedure days, for appearance, activity, food and water intake, body weight, wound condition, and signs of pain or distress. Humane endpoints included persistent anorexia, inability to access food or water, severe lethargy or recumbency, dyspnea, self-injury, uncontrolled pain or distress, and severe wound complications. No additional postoperative analgesia was administered; animals were closely monitored for pain or distress. At the experimental endpoint, animals were euthanized with an intraperitoneal overdose of pentobarbital sodium (>150 mg/kg).

### Gentamicin administration

For postauricular administration, the body-weight-adjusted dose was injected into the right postauricular sulcus. For intramuscular administration, the same nominal dose was injected into the right thigh muscle. In the repeated-dose experiment, gentamicin was administered at 200 mg/kg per injection every 12 h for 7 consecutive days, for a total of 14 injections. For intratympanic injection, animals were anesthetized with 1% pentobarbital sodium (30 mg/kg). A 5–8-mm postauricular incision was made superior to the tympanic bulla, followed by blunt dissection to expose the bulla. After identification of the facial nerve, a 2–3-mm posterior tympanotomy was created posterior to the stylomastoid foramen using fine forceps. Gentamicin was administered at a nominal dose of 200 mg/kg using a 300 mg/mL solution. The volume was slowly introduced into the tympanic cavity through the posterior tympanotomy under microscopic visualization. This procedure is hereafter referred to as intratympanic injection. The administered volume was anatomically feasible because the guinea pig tympanic cavity has an estimated anatomical volume of approximately 287 µL, and previous experimental studies have used 150–200 µL to achieve near-complete or complete filling of the tympanic bulla (Daoudi et al., 2025; Mikulec et al., 2009). The tympanotomy was sealed with fascia, and the postauricular incision was sutured.

### ABR recording

ABR testing was performed in the untreated control group and in animals receiving repeated postauricular or intramuscular gentamicin injections. Both ears were tested before treatment, 1 day after the final injection, and 7 days after the final injection. ABR testing was performed on anesthetized animals in a shielded room, as detailed in Ding et al. (2020). Needle electrodes were placed subcutaneously: one at the head midline for recording, one in the ipsilateral earlobe for reference, and one in the contralateral earlobe for grounding. The TDT III system and RZ6 recorder were used for ABR. Tone bursts at 1, 2, 4, 6, and 8 kHz were delivered *via* an ER2 earphone. Each burst had a 0.5 ms rise-fall time and a 3 ms plateau. Stimuli were presented at 20/s with a 10 ms recording window. Signals were amplified, filtered (100–3,000 Hz), and averaged. Thresholds were determined by reducing stimulus intensity in 10 dB steps from 90 dB SPL. If no waveform was detected at 90 dB, the threshold was set at 100 dB SPL.

### Evaluation of effects of gentamicin on body weight and renal function

Body weight was recorded daily throughout the repeated-dose experiment and for 7 days after the final injection. Seven days after the final injection, blood was collected by cardiac puncture. Samples were allowed to stand for 1 h at room temperature and were centrifuged at 14,000 rpm for 15 min. Serum creatinine (Scr) and blood urea nitrogen (BUN) were measured as indices of renal function (Udupa & Prakash, 2019). These assessments included the untreated control and repeated-dose postauricular and intramuscular groups.

### Surface preparation of cochlear basilar membrane

Cochlear hair cell morphology was evaluated in two experimental cohorts. The repeated-dose cohort included the untreated control group, the ipsilateral and contralateral ears of the postauricular injection group, and the intramuscular injection group. Cochleae were collected 7 days after the final injection. The single-dose cohort included the intratympanic, postauricular, and intramuscular injection groups, with cochleae collected at 2.5 or 12.5 h after injection. Under deep anesthesia, the guinea pigs were decapitated after sequential transcardiac infusion of cold phosphate buffer saline and 10% formaldehyde in PBS. The temporal bone was removed and immersed in fixative overnight. After fixation, the entire cochlear basilar membrane was micro-dissected out in PBS (Ding et al., 2003, 1981). Subsequently, the cochlear basilar membranes were incubated in 1% Triton-100 in PBS (PBST) for 30 min and in a blocking medium (10% donkey or goat serum in 0.1 M PBS) for 1 h at room temperature. Afterwards, the basilar membranes were incubated with a fluorescently labeled phalloidin antibody (Cat#YP0053S, 1:200; Bioscience, Allentown, PA, USA) for 2 h. After rinsing with 0.5% PBST, specimens were mounted in glycerin on glass slides coverslipped as flat surface preparations (Zhang et al., 2019). The samples were imaged using a Zeiss LSM 880 microscope. A z-stack series was utilized to ensure that the entire basilar membranes were photographed completely.

### Localization and quantification of cochlear hair cells

**Cochleograms were constructed as previously described** (Ding et al., 2021, 2003; McFadden et al., 2005). OHC were counted every 0.24 mm along the membrane using a 400x fluorescent microscope, considering cells present if their stereocilia and cuticular plate were intact, and absent if a phalangeal scar was visible. These counts were input into cochleogram software to compare with lab standards based on data from six healthy albino guinea pigs. The resulting cochleograms display the percentage of missing hair cells by cochlear location, normalized to the distance from the apex. Averaged cochleograms were created for each experimental group using the software, and the data was further analyzed and compared using Excel and GraphPad Prism.

### Perilymph gentamicin measurement

Perilymph was collected terminally from the single-dose surgical middle-ear, postauricular, and intramuscular groups at 2.5 and 12.5 h after administration. These time points were selected to represent earlier and later phases of inner-ear exposure based on prior reports of slow entry and prolonged retention of gentamicin in perilymph. Before perilymph collection from the intratympanic group, all visible residual solution in the tympanic cavity was carefully removed using sterile absorbent paper. Sampling was initiated only after no visible liquid remained. Perilymph was subsequently aspirated using a clean flattened needle through the round- and oval-window regions and stored at −80 °C until analysis.

LC–MS/MS was performed by EverPro Medical Co., Ltd. (Changsha, China) using a Shimadzu Prominence LC-20ADXR UFLC system coupled to an AB Sciex API 5000 triple-quadrupole mass spectrometer. Separation was achieved on a Welch Ultimate XB-C18 column (150 mm × 4.6 mm, 5 μm) using 0.1% formic acid in water (mobile phase A) and acetonitrile (mobile phase B). The proportion of B was held at 10% for 0.50 min, increased to 72% at 2.00 min and 95% at 2.01 min, held until 4.00 min, and returned to 10% at 4.01 min. The total run time was 6.50 min, the flow rate was 0.600 mL/min, and the column temperature was 40 °C. Data were acquired in multiple-reaction-monitoring mode. The source temperature was 550 °C and the ion-spray voltage was 5,500 V. Nitrogen nebulizing and auxiliary gases were each set at 55 arbitrary units.

### Statistical analysis

Descriptive data are presented as the means and standard deviations (SDs). Hair cell survival, body weight, and serum biochemical indicators were compared using one-way analysis of variance (ANOVA) followed by Tukey’s multiple comparisons test. Normality of the data distribution was assessed using the Shapiro–Wilk test, and homogeneity of variance was evaluated using Levene’s test. If these assumptions were not satisfied, appropriate nonparametric tests were planned. The ABR threshold data were analyzed using a linear mixed-effects model. ABR threshold shifts from baseline were treated as the dependent variable. Prespecified pairwise comparisons between the postauricular and intramuscular groups at the corresponding time points and frequencies were performed with Šidák adjustment for multiple comparisons. Because potential ipsilateral-contralateral differences were particularly relevant to the postauricular route, an additional mixed-effects analysis was performed within the postauricular group. All the statistical analyses were performed using GraphPad Prism software (version 9.0.0) (San Diego, California, USA, www.graphpad.com), and the level of statistical significance was set at *P* < 0.05.

## Results

### ABR threshold elevation after repeated postauricular and intramuscular administration

Baseline ABR thresholds were within the expected range in the untreated control, postauricular, and intramuscular groups (Fig. 1A). One day after the final gentamicin injection, thresholds were elevated by approximately 30–40 dB across the tested frequencies in both repeated-dose groups relative to baseline (Fig. 1B). Seven days after the final injection, thresholds had increased further, reaching approximately 60–70 dB above baseline at several frequencies, with the most pronounced deficits at the higher frequencies (Fig. 1C). No significant difference was detected between the postauricular and intramuscular routes or between the ipsilateral and contralateral ears within the postauricular group at the corresponding assessment times.

**Figure 1:**
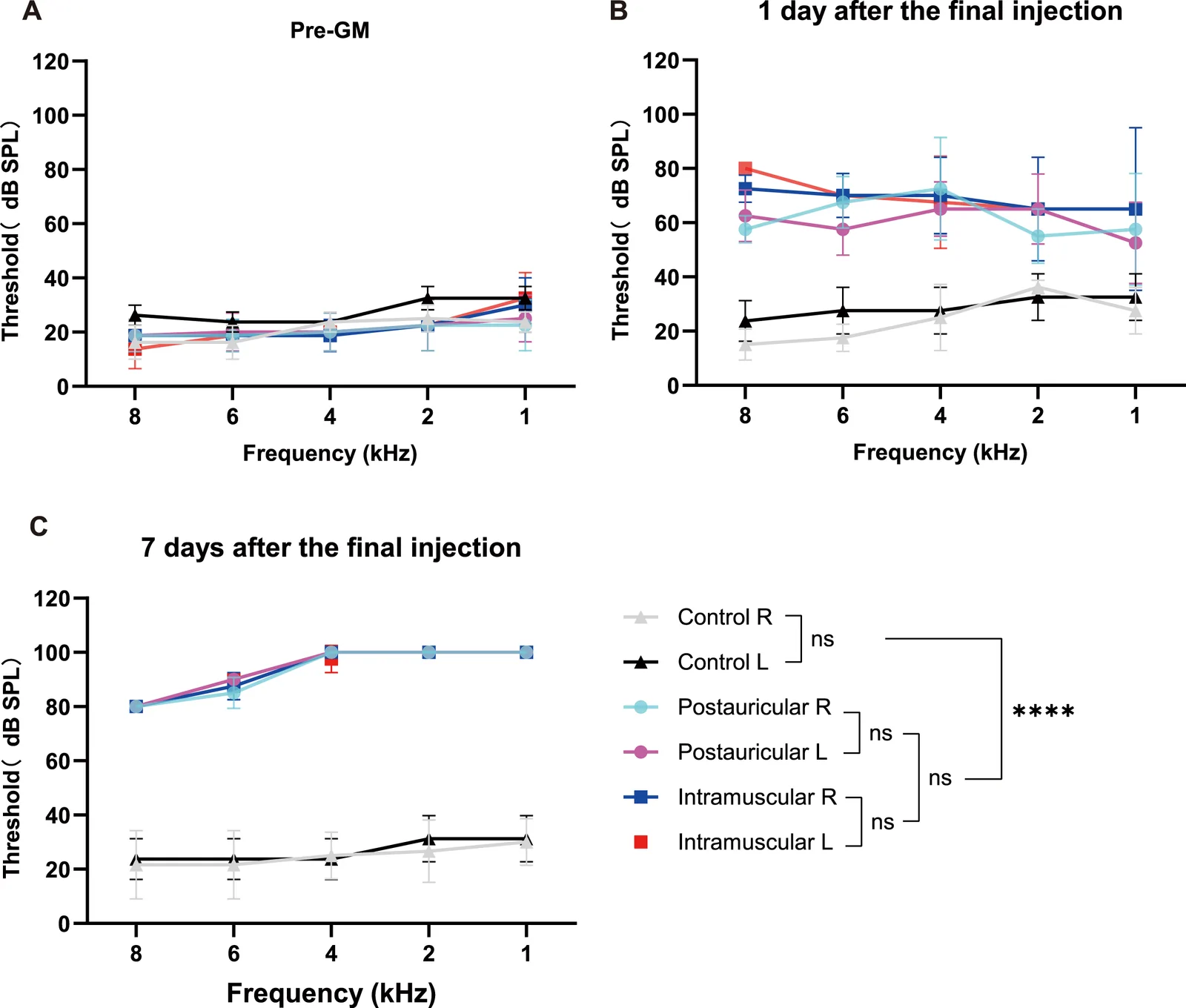
Auditory brainstem response thresholds following repeated postauricular or intramuscular gentamicin administration. Auditory brainstem response (ABR) thresholds were measured in the untreated control, postauricular, and intramuscular groups before gentamicin administration (A), 1 day after the final injection (B), and 7 days after the final injection (C). Guinea pigs in the two treatment groups received gentamicin at 200 mg/kg per injection every 12 h for 7 consecutive days, for a total of 14 injections. Thresholds were assessed separately in the right (R) and left (L) ears at 1, 2, 4, 6, and 8 kHz. A value of 100 dB SPL was assigned when no reproducible waveform was detected at the maximum tested stimulus level of 90 dB SPL. Data are presented as the mean ± SD. ABR threshold shifts from baseline were analyzed using a linear mixed-effects model, with prespecified pairwise comparisons adjusted using the Šidák method. Both gentamicin-treated groups showed significantly elevated ABR thresholds compared with the untreated control 7 days after the final injection (****P < 0.0001). No significant differences were detected between postauricular and intramuscular administration or between the ipsilateral and contralateral ears within the postauricular group. ns, not significant; GM, gentamicin.

### OHC loss after repeated postauricular and intramuscular administration

In order to determine whether repeated postauricular and multiple intramuscular injections of GM cause the equal degree of damage to cochlear hair cells, the hair cell density in the cochlea were measured. Seven days after the final injection, extensive OHC loss was present in the repeated-dose postauricular and intramuscular groups, whereas inner hair cells were comparatively preserved at this endpoint (Fig. 2). The averaged cochleograms showed broadly overlapping OHC-loss patterns in the ipsilateral and contralateral ears of the postauricular group and in the intramuscular group (Fig. 3). No significant between-route difference in the measured OHC-loss outcome was detected.

**Figure 2:**
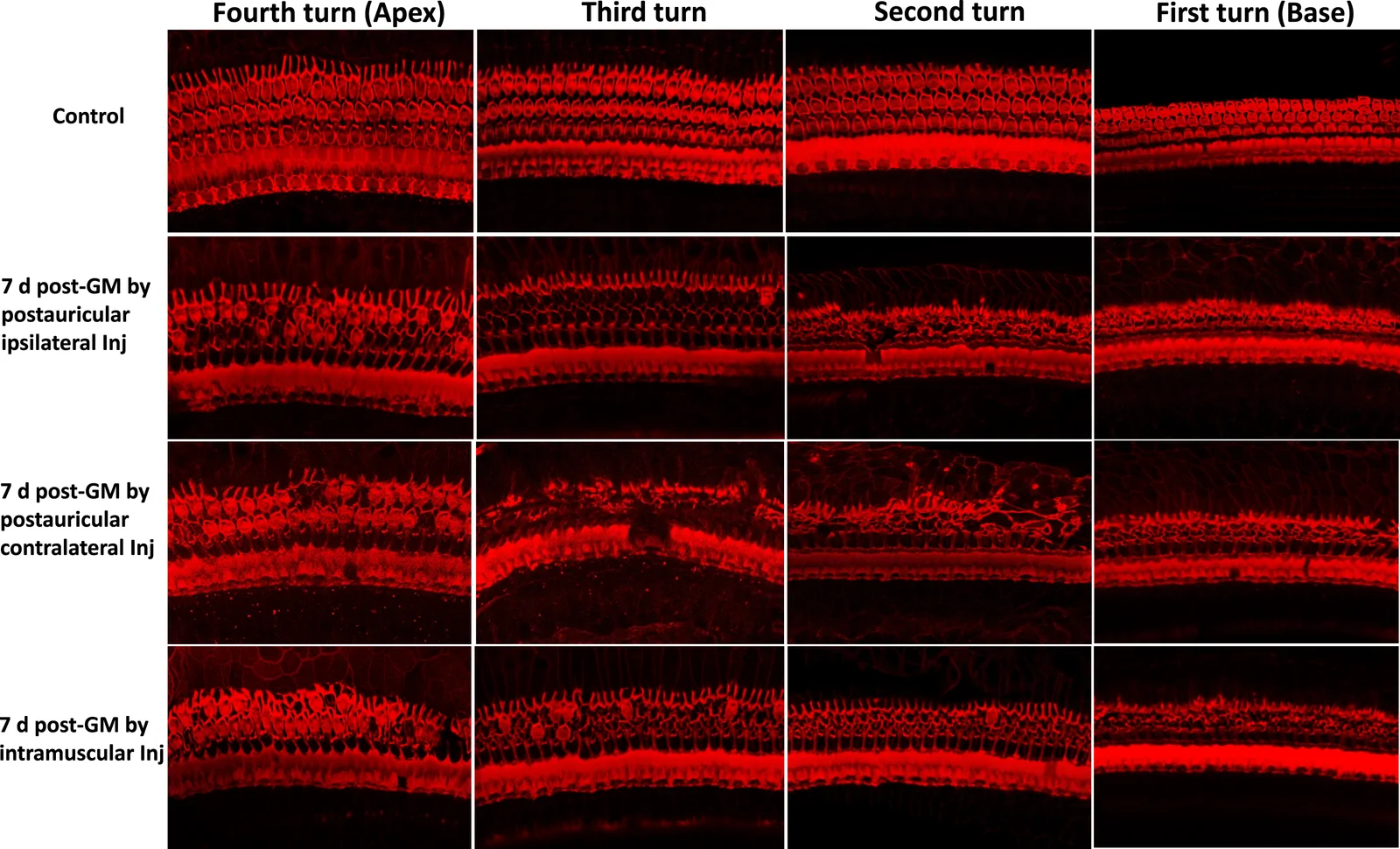
Cochlear outer hair cell loss following repeated postauricular or intramuscular gentamicin administration. Representative confocal images of phalloidin-labeled cochlear surface preparations obtained from the untreated control group, the ipsilateral and contralateral ears of the postauricular group, and the intramuscular group 7 days after the final gentamicin injection. Guinea pigs in the treatment groups received gentamicin at 200 mg/kg per injection every 12 h for 7 consecutive days, for a total of 14 injections. Images are arranged from the fourth cochlear turn at the apex to the first turn at the base. Extensive outer hair cell loss was observed throughout the cochlea in both ears of the postauricular group and in the intramuscular group, whereas inner hair cells were comparatively preserved. OHC, outer hair cell; GM, gentamicin.

**Figure 3:**
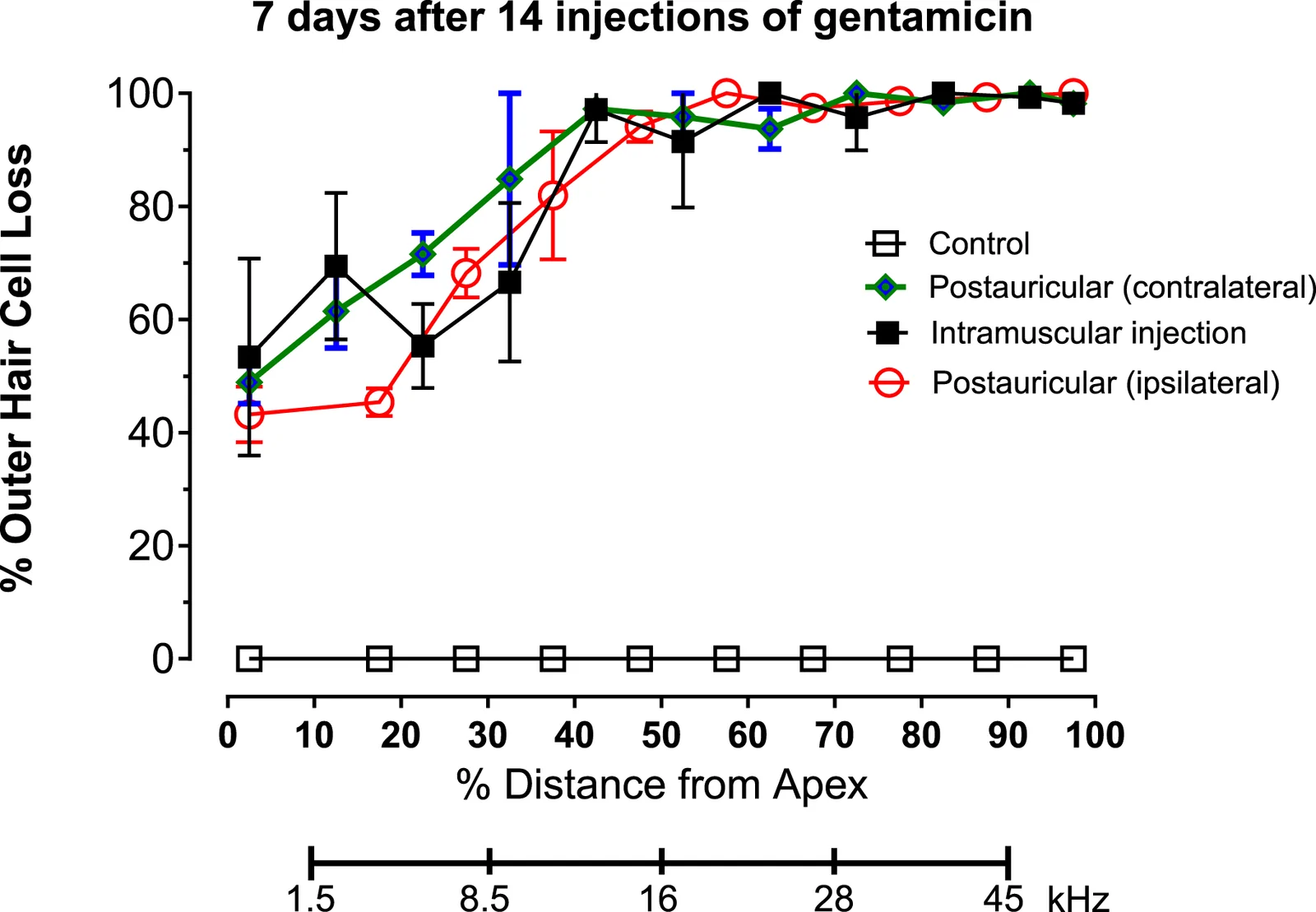
Cochleographic distribution of outer hair cell loss following repeated gentamicin administration. The percentage of outer hair cell loss is plotted as a function of percentage distance from the cochlear apex in the untreated control group, the ipsilateral and contralateral ears of the postauricular group, and the intramuscular group. Cochleae were collected 7 days after the final injection of a 14-injection gentamicin regimen. Approximate frequency locations along the guinea pig cochlea are indicated beneath the horizontal axis. Data are presented as the mean ± SD. The postauricular ipsilateral, postauricular contralateral, and intramuscular groups showed broadly overlapping patterns of outer hair cell loss, with progressively greater damage toward the basal region. No significant between-route difference in the measured outer hair cell loss was detected. OHC, outer hair cell.

### Effects of multiple administration of gentamicin in postauricular and intramuscular injections on body weight and renal function in experimental animals

In order to explore the effects of different routes of gentamicin administration on the body weight of experimental animals, the body weight of animals was tested daily until the end of the experiment. Control animals gained weight during the observation period, whereas body weight declined progressively in both gentamicin-treated groups (Figs. 4A, 4B). At the end of follow-up, body weight differed significantly between each gentamicin-treated group and the control group, but not between the postauricular and intramuscular groups. This indicates that repeated postauricular and intramuscular injections of gentamicin caused similar degree of weight loss in the guinea pigs.

**Figure 4:**
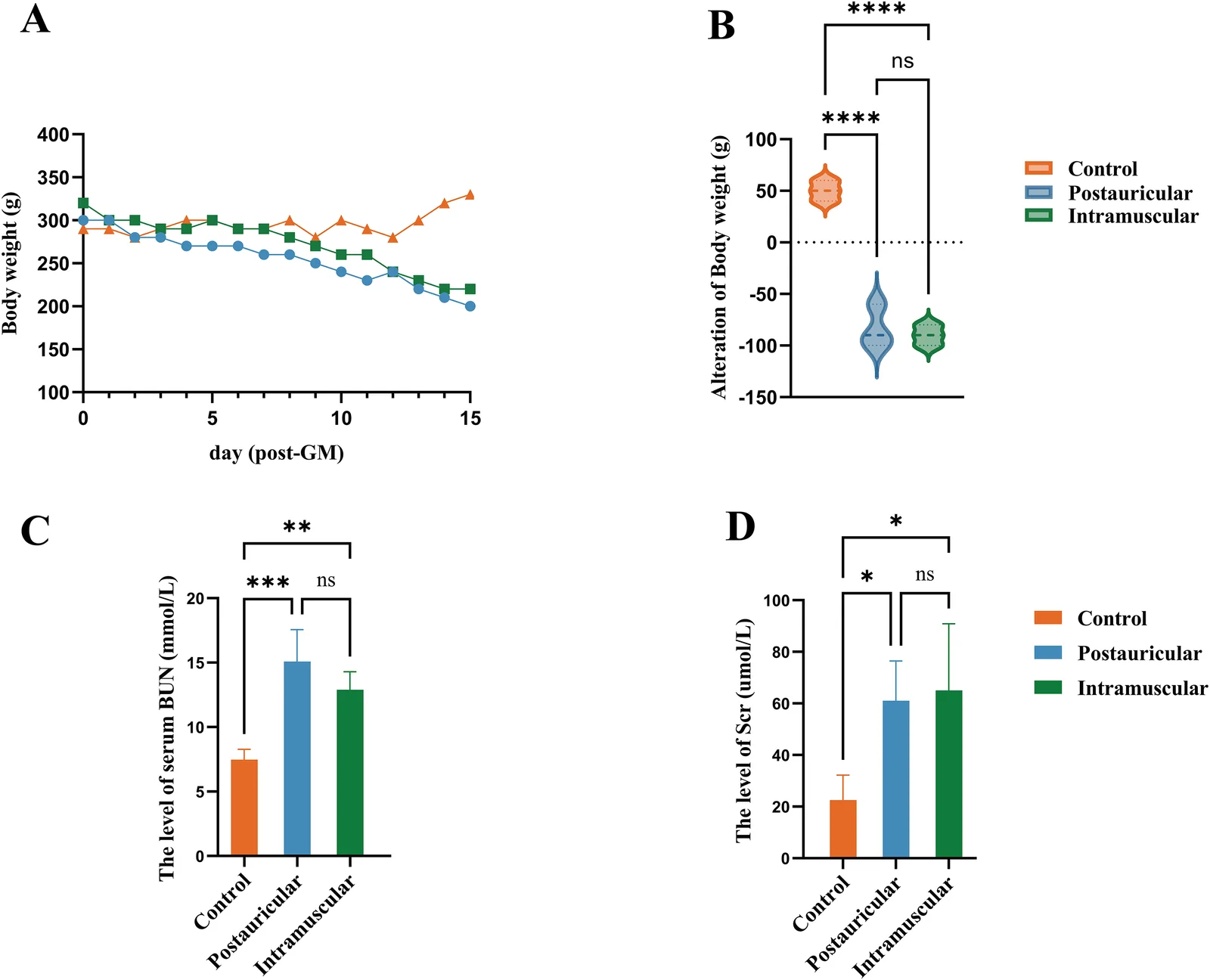
Changes in body weight and renal-function indices following repeated gentamicin administration. (A) Daily body weight during the repeated-dose experiment and the 7-day period following the final injection. Guinea pigs in the postauricular and intramuscular groups received gentamicin at 200 mg/kg per injection every 12 h for 7 consecutive days. (B) Change in body weight from baseline to the end of the observation period. (C) Serum blood urea nitrogen (BUN) and (D) serum creatinine (Scr) concentrations measured 7 days after the final injection. Data are presented as the mean ± SD. Comparisons were performed using one-way analysis of variance followed by Tukey’s multiple-comparison test. Both gentamicin-treated groups showed greater body-weight reduction and higher serum BUN and Scr concentrations than the untreated control group, whereas no significant differences were detected between the postauricular and intramuscular groups. *P < 0.05, **P < 0.01, ***P < 0.001, and ****P < 0.0001; ns, not significant; GM, gentamicin.

The serum blood urea nitrogen (BUN) and serum creatinine (Scr) were examined on the 7 days after the final dose of gentamicin. Serum BUN and Scr measured 7 days after the final injection were elevated in both gentamicin-treated groups compared with the untreated control (Figs. 4C, 4D). No significant difference was detected between the postauricular and intramuscular groups.

### OHC morphology after a single administration

To demonstrate whether a single injection of gentamicin *via* three different routes (intratympanic, postauricular, and intramuscular) led to the destruction of cochlear hair cells, the cochlear basilar membrane of the test animals was prepared as flat surface preparations for the determination of hair cell density. At 2.5 h after a single gentamicin injection, OHC survival was 100% in all examined animals in the intratympanic, postauricular, and intramuscular groups, indicating that no morphologically detectable loss of OHC cuticular plates was present at this time point (Fig. S1). At 12.5 h, outer hair cell loss was observed in the intratympanic group, whereas no cuticular-plate loss was detected after postauricular or intramuscular administration (Fig. 5).

**Figure 5:**
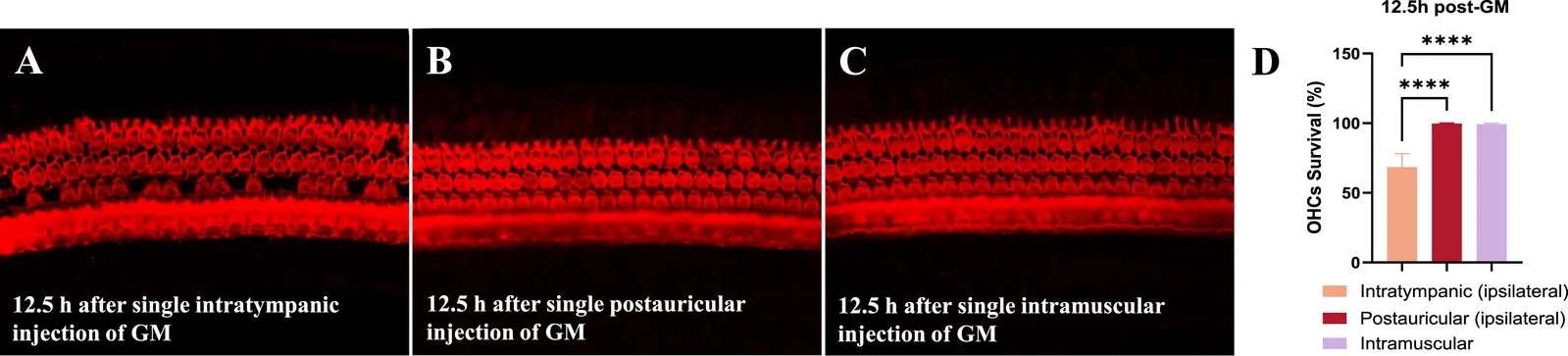
Cochlear outer hair cell morphology 12.5 h after a single gentamicin administration. Representative phalloidin-labeled cochlear surface preparations obtained 12.5 h after a single intratympanic injection through a surgically created posterior tympanotomy (A), postauricular injection (B), or intramuscular injection (C). All animals received a nominal gentamicin dose of 200 mg/kg. For intratympanic injection, a 300 mg/mL gentamicin solution was administered through the posterior tympanotomy; a typical 300-g guinea pig received 60 mg in a volume of 0.20 mL. Moderate outer hair cell cuticular-plate loss was observed in the basal turn after intratympanic injection, whereas no morphologically detectable loss was observed after postauricular or intramuscular injection. (D) Quantification of outer hair cell survival in the examined basal-turn region. Data are presented as the mean ± SD. Comparisons were performed using one-way analysis of variance followed by Tukey’s multiple-comparison test. Outer hair cell survival was significantly lower in the intratympanic group than in either the postauricular or intramuscular group (****P < 0.0001). OHC, outer hair cell; GM, gentamicin.

### Perilymph gentamicin concentrations after a single administration

At 2.5 h, perilymph gentamicin concentrations were 4,103 ± 349 μg/mL in the intratympanic group, 18.43 ± 4.70 μg/mL in the postauricular group, and 21.84 ± 16.75 μg/mL in the intramuscular group. The concentration was significantly higher after intratympanic administration than after either postauricular or intramuscular administration (both *P* < 0.0001), whereas no significant difference was detected between the postauricular and intramuscular groups (*P* > 0.05; Fig. 6A).

**Figure 6:**
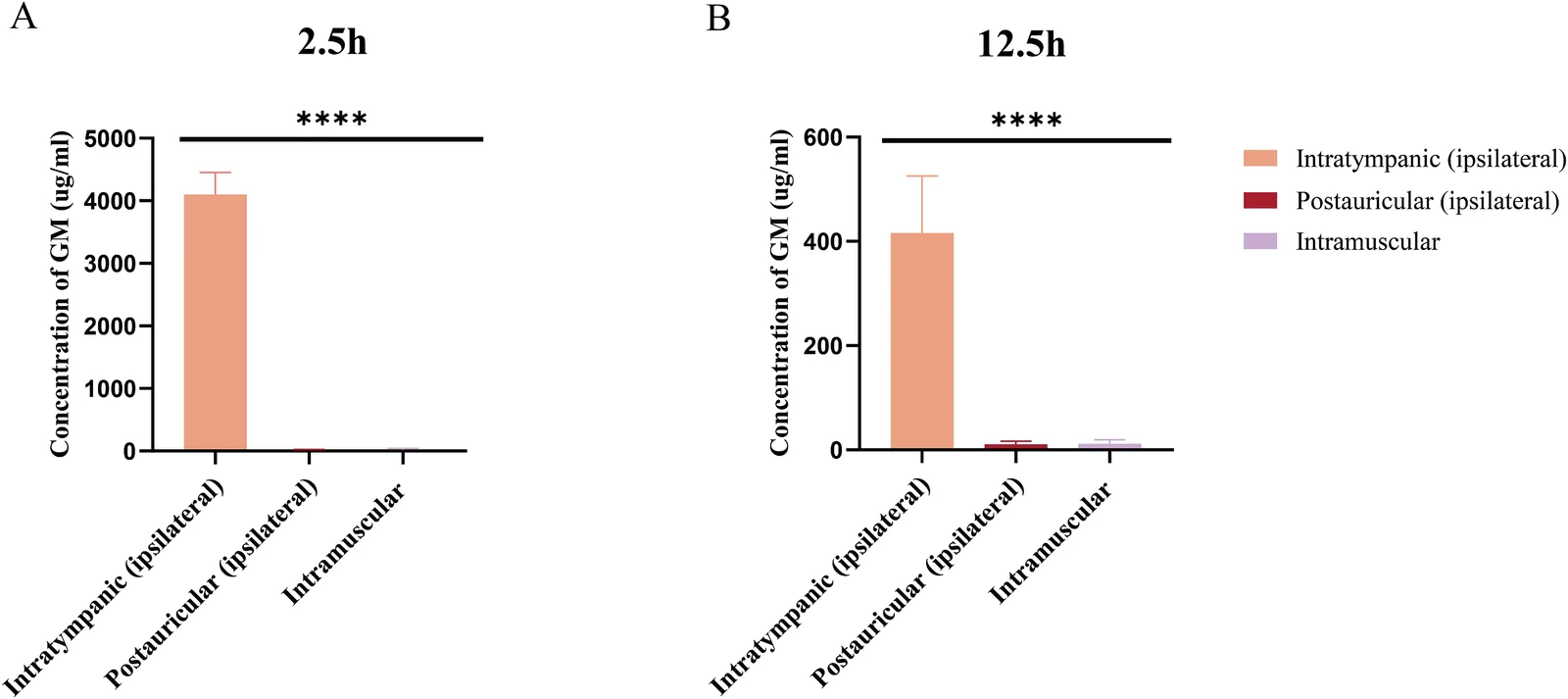
Perilymph gentamicin concentrations after a single administration by different routes. Perilymph gentamicin concentrations were measured 2.5 h (A) and 12.5 h (B) after a single intratympanic, postauricular, or intramuscular administration. Bars represent the mean ± SD. Comparisons were performed separately at each time point using one-way analysis of variance followed by Tukey’s multiple-comparison test. Intratympanic administration produced significantly higher perilymph gentamicin concentrations than either postauricular or intramuscular administration at both time points (all ****P < 0.0001). No significant difference was detected between postauricular and intramuscular administration at 2.5 or 12.5 h (adjusted (*P* > 0.999) at both time points). Note the discontinuous vertical axes. GM, gentamicin.

At 12.5 h, the respective concentrations were 416.20 ± 109.10, 10.86 ± 5.98, and 11.58 ± 7.88 μg/mL. Intratympanic administration again produced significantly higher concentrations than the other two routes (both *P* < 0.0001), and the postauricular and intramuscular groups did not differ significantly (*P* > 0.05; Fig. 6B). These two terminal measurements demonstrate substantially greater perilymph exposure after direct middle-ear delivery.

## Discussion

Due to the unique anatomy of the blood–labyrinth barrier, drug delivery is the key determinant of clinical effectiveness in patients with inner ear diseases. This study compared postauricular, intramuscular, and intratympanic administration using functional, histological, systemic-effect, and perilymph-concentration outcomes. A single postauricular injection did not produce higher perilymph gentamicin concentrations or greater OHC loss than a single intramuscular injection at the two sampled time points. Repeated postauricular and intramuscular administration produced comparable bilateral ABR threshold elevation, OHC loss, body-weight reduction, and renal dysfunction. Collectively, these observations provide no evidence that postauricular administration preferentially delivered gentamicin to the inner ear relative to intramuscular administration under the present conditions.

### Anatomical and pharmacological considerations for postauricular drug delivery

Postauricular injection is a local drug delivery method that has emerged in a few countries in recent years. Proponents believe that drugs injected behind the ear can enter the capillary network of the ear through local tissue absorption, and then flow into the sigmoid sinus and endolymphatic sac (Chen et al., 2022). They proposed that drugs injected into the subcutaneous tissue behind the ear can enter the cochlea and directly act on the hair cells (Yang, Yu & Ma, 2007). These anatomical possibilities should not be dismissed, but neither do they independently demonstrate drug passage into the perilymph or target tissues.

An operationally meaningful local pathway should yield evidence of preferential exposure, such as higher ipsilateral perilymph concentration, greater ipsilateral tissue accumulation, faster inner-ear appearance, or a higher inner-ear-to-plasma exposure ratio than a matched systemic route. The single-dose gentamicin measurements did not show greater perilymph exposure after postauricular than after intramuscular administration, and repeated postauricular administration produced bilateral rather than predominantly ipsilateral injury. These results do not support a functionally important preferential local pathway for gentamicin in the current protocol, although a small, transient, or drug-specific local component cannot be excluded.

Methylene blue and fluorescently labeled compounds have been used to investigate distribution after postauricular administration (Chen et al., 2022; Huang, Zhao & Wang, 2020). However, detection of a tracer in or around the inner ear does not, by itself, establish the route by which it reached that location. A tracer may arrive after systemic absorption or may spread locally through soft tissue, lymphatic, perivascular, or regional vascular connections. Thus, tracer staining may be compatible with a local distribution component but cannot distinguish it from systemic delivery.

Conversely, qualitative staining in inner-ear vessels or tissues does not demonstrate passage across the BLB, a pharmacologically relevant perilymph concentration, or delivery of the intact parent drug. Methylene blue and fluorescent conjugates also differ from gentamicin and glucocorticoids in size, charge, protein binding, and metabolism. Definitive pathway characterization requires quantitative spatial tracing of an appropriately labeled therapeutic molecule together with local-tissue, ipsilateral-perilymph, and contralateral-perilymph measurements.

In the present study, postauricular administration did not produce higher perilymph gentamicin concentrations than intramuscular administration at either 2.5 or 12.5 h. Moreover, repeated postauricular administration produced bilateral rather than clearly ipsilateral-predominant cochlear injury. These observations do not support a major preferential local delivery pathway for gentamicin under the conditions tested. Nevertheless, because complete plasma and perilymph concentration–time profiles were not obtained, a small or temporally restricted local contribution cannot be excluded.

### Interpretation of the gentamicin findings

Systemically administered gentamicin enters the inner-ear fluids relatively slowly and is cleared from the inner ear more slowly than from the systemic circulation (Ding et al., 2003; Federspil, Schiitzle & Tiesler, 1976; Stupp, 1970). This pharmacokinetic behavior is important for interpreting both the single-dose and repeated-dose findings in the present study.

After a single administration, perilymph gentamicin concentrations at 2.5 h were 4,103 ± 349 μg/mL after intratympanic injection, compared with 18.43 ± 4.70 μg/mL after postauricular injection and 21.84 ± 16.75 μg/mL after intramuscular injection. At 12.5 h, the corresponding concentrations were 416.20 ± 109.10, 10.86 ± 5.98, and 11.58 ± 7.88 μg/mL, respectively. The markedly higher concentrations after intratympanic injection are consistent with direct exposure of the round-window region and subsequent entry into the inner ear. In contrast, no significant difference was detected between postauricular and intramuscular administration at either sampled time point. Therefore, these two measurements provide no evidence that postauricular administration increased gentamicin delivery to the perilymph relative to the intramuscular route.

The morphological findings were consistent with the concentration differences. No morphologically detectable outer hair cell loss was observed at 2.5 h after any administration route, whereas outer hair cell loss was evident at 12.5 h after intratympanic injection. Importantly, absence of morphologically detectable hair cell loss at an early time point does not imply absence of cellular drug uptake or early subcellular injury. Hiel et al. (1993) demonstrated that gentamicin accumulates in cochlear sensory hair cells before measurable hearing impairment becomes evident (Cheng et al., 2023; Hiel et al., 1993).

These findings also provide a plausible explanation for the progressive ABR threshold elevation observed between 1 and 7 days after the final injection in the repeated-dose experiment. Aminoglycoside ototoxicity may continue to evolve after systemic administration has ceased. Thus, the delayed deterioration observed in the present study is compatible with a temporal dissociation between gentamicin uptake and the subsequent manifestation of functional and structural cochlear injury. Repeated postauricular and intramuscular administration produced broadly similar bilateral patterns of ABR threshold elevation and outer hair cell loss. Comparable renal-function changes were also observed between the two groups. These findings are consistent with a substantial systemic component after postauricular administration.

### Drug-specific considerations: cisplatin and glucocorticoids

Qiu et al. (2022) compared postauricular and intraperitoneal administration of cisplatin in guinea pigs. Postauricular administration produced greater cochlear hair cell damage in the ipsilateral ear than systemic administration, suggesting a possible local component; however, substantial contralateral cochlear damage, body-weight loss, and renal toxicity were also observed. The authors therefore characterized postauricular administration as a hybrid route with both topical and systemic features (Qiu et al., 2022). This study is important because it indicates that postauricular administration cannot necessarily be classified as exclusively local or exclusively systemic, and that the relative contribution of each component may vary according to the drug studied. Our gentamicin findings differ from the cisplatin observations in that no preferential increase in perilymph gentamicin concentration was detected after postauricular administration relative to intramuscular administration at either sampled time point. This difference further emphasizes that the delivery characteristics of individual drugs should be evaluated directly rather than inferred from studies using other compounds.

Glucocorticoids require similar caution. Systemically administered corticosteroids can reach the inner-ear fluids; thus, the BLB restricts rather than completely prevents their entry. In guinea pigs, Parnes, Sun & Freeman (1999) demonstrated measurable cochlear-fluid concentrations after systemic corticosteroid administration, while substantially higher inner-ear concentrations were achieved after topical middle-ear administration. Similar findings have subsequently been reported in humans: intratympanic methylprednisolone produced higher cochlear perilymph exposure than intravenous administration (Bird et al., 2007, 2011), and intratympanic dexamethasone resulted in markedly higher perilymph concentrations and lower plasma concentrations than intravenous administration (Bird et al., 2011). More recent studies further indicate that inner-ear glucocorticoid exposure is strongly influenced by the route of administration, formulation, and local cochlear conditions, including inflammation or surgical disruption of the blood–labyrinth barrier (He et al., 2024; Ke et al., 2024).

Accordingly, the present results indicate that postauricular administration did not provide preferential gentamicin delivery to the perilymph relative to intramuscular administration at the sampled time points, but they do not exclude the possibility that other drugs, formulations, or pathological conditions may behave differently.

### Limitations

Several limitations should be acknowledged. First, pharmacokinetic assessment was limited to two time points after single-dose administration, and plasma and perilymph concentrations were not measured during the repeated-dose protocol. Therefore, complete concentration–time profiles and pharmacokinetic equivalence between postauricular and intramuscular administration could not be established. Second, route-matched vehicle controls and a surgical sham control were not included, and the intratympanic injection used as the local-delivery comparator was more invasive than conventional clinical intratympanic injection. Third, only guinea pigs and gentamicin were investigated. Species-specific anatomical differences and drug-specific physicochemical and pharmacokinetic properties may limit the generalizability of these findings to humans or to other drugs and formulations.

Future studies incorporating more comprehensive plasma–perilymph pharmacokinetic sampling, appropriate route-matched controls, and additional drug classes will be needed to further characterize the contribution of systemic and local pathways after postauricular administration.

## Conclusions

In this guinea pig model, postauricular gentamicin administration did not provide preferential access to the inner ear compared with intramuscular administration. The observed cochlear and systemic effects were consistent with predominantly systemic exposure.

## Supplemental Information

10.7717/peerj.21796/supp-1 Supplemental Information 1 Data.

10.7717/peerj.21796/supp-2 Supplemental Information 2 ARRIVE Checklist.

10.7717/peerj.21796/supp-3 Supplemental Information 3 Cochlear outer hair cell morphology at 2.5 h after a single gentamicin injection. Representative cochlear surface preparations from the intratympanic, postauricular, and intramuscular groups are shown in panels A–C. D shows OHC survival in the examined cochlear regions. OHC survival was 100% in all animals in each group; No morphologically detectable loss of OHC cuticular plates was observed at 2.5 h.

## Additional Information and Declarations

### Data Availability

The following information was supplied regarding data availability:

The raw measurements are available in the Supplemental Files.
