# Supplementary material for: Postauricular gentamicin administration does not provide preferential inner-ear delivery in guinea pigs
Source: PeerJ. 2026 Oct 27;14:e21796. doi: 10.7717/peerj.21796 (PMC13721883; doi:10.7717/peerj.21796)
Supplement: Supplementary file 1 [file peerj-14-21796-s002.pdf]

NOTE: Please save this file locally before filling in the table, DO NOT work on the file within your internet browser as changes will not be saved. Adobe Acrobat Reader (available free here) is recommended for completion.

The ARRIVE guidelines 2.0: author checklist

The ARRIVE Essential 10

These items are the basic minimum to include in a manuscript. Without this information, readers and reviewers cannot assess the reliability of the findings.

Item		Recommendation	Section/line number, or reason for not reporting
**Study design**	1	For each experiment, provide brief details of study design including:	
		a. The groups being compared, including control groups. If no control group has been used, the rationale should be stated.	Materials and Methods, Lines 76 – 116; Table 1.
		b. The experimental unit (e.g. a single animal, litter, or cage of animals).	Materials and Methods, Lines 99 – 100.
**Sample size**	2	a. Specify the exact number of experimental units allocated to each group, and the total number in each experiment. Also indicate the total number of animals used.	Materials and Methods, Lines 78 – 81; Table 1.
		b. Explain how the sample size was decided. Provide details of any *a priori* sample size calculation, if done.	Materials and Methods, Lines 79 – 82; Table 1.
**Inclusion and exclusion criteria**	3	a. Describe any criteria used for including and excluding animals (or experimental units) during the experiment, and data points during the analysis. Specify if these criteria were established *a priori*. If no criteria were set, state this explicitly.	Materials and Methods, Lines 78 – 82, 96 – 99.
		b. For each experimental group, report any animals, experimental units or data points not included in the analysis and explain why. If there were no exclusions, state so.	Materials and Methods, Line 81.
		c. For each analysis, report the exact value of *n* in each experimental group.	Table 1; Materials and Methods, Lines 106 – 107.
**Randomisation**	4	a. State whether randomisation was used to allocate experimental units to control and treatment groups. If done, provide the method used to generate the randomisation sequence.	Not reported.
		b. Describe the strategy used to minimise potential confounders such as the order of treatments and measurements, or animal/cage location. If confounders were not controlled, state this explicitly.	Not reported.
**Blinding**	5	Describe who was aware of the group allocation at the different stages of the experiment (during the allocation, the conduct of the experiment, the outcome assessment, and the data analysis).	Not reported.
**Outcome measures**	6	a. Clearly define all outcome measures assessed (e.g. cell death, molecular markers, or behavioural changes).	Materials and Methods, Lines 118 – 209.
		b. For hypothesis-testing studies, specify the primary outcome measure, i.e. the outcome measure that was used to determine the sample size.	Not explicitly reported.
**Statistical methods**	7	a. Provide details of the statistical methods used for each analysis, including software used.	Materials and Methods, Lines 201 – 209.
		b. Describe any methods used to assess whether the data met the assumptions of the statistical approach, and what was done if the assumptions were not met.	Materials and Methods, Lines 204 – 207.
**Experimental animals**	8	a. Provide species-appropriate details of the animals used, including species, strain and substrain, sex, age or developmental stage, and, if relevant, weight.	Materials and Methods, Lines 78 – 79.
		b. Provide further relevant information on the provenance of animals, health/immune status, genetic modification status, genotype, and any previous procedures.	Materials and Methods, Lines 79, 82 – 83.
**Experimental procedures**	9	For each experimental group, including controls, describe the procedures in enough detail to allow others to replicate them, including:	Materials and Methods, Lines 109 – 199.
		a. What was done, how it was done and what was used.	Materials and Methods, Lines 127 – 128, 131 – 142, 149 – 152, 178 – 182.
		b. When and how often.	Materials and Methods, Lines 84 – 90, 119, 185 – 199.
		c. Where (including detail of any acclimatisation periods).	
		d. Why (provide rationale for procedures).	Materials and Methods, Lines 84 – 90, 119, 185 – 199.
**Results**	10	For each experiment conducted, including independent replications, report:	
		a. Summary/descriptive statistics for each experimental group, with a measure of variability where applicable (e.g. mean and SD, or median and range).	Results, Lines 211 – 304; Statistical analysis, Lines 201 – 203.
		b. If applicable, the effect size with a confidence interval.	Not reported.

The Recommended Set

These items complement the Essential 10 and add important context to the study. Reporting the items in both sets represents best practice.

Item		Recommendation	Section/line number, or reason for not reporting
**Abstract**	11	Provide an accurate summary of the research objectives, animal species, strain and sex, key methods, principal findings, and study conclusions.	Abstract, Lines 18 – 31.
**Background**	12	a. Include sufficient scientific background to understand the rationale and context for the study, and explain the experimental approach.	Introduction, Lines 35 – 74.
		b. Explain how the animal species and model used address the scientific objectives and, where appropriate, the relevance to human biology.	Introduction, Lines 71 – 74; Materials and Methods, Lines 78 – 79.
**Objectives**	13	Clearly describe the research question, research objectives and, where appropriate, specific hypotheses being tested.	Introduction, Lines 71 – 74.
**Ethical statement**	14	Provide the name of the ethical review committee or equivalent that has approved the use of animals in this study, and any relevant licence or protocol numbers (if applicable). If ethical approval was not sought or granted, provide a justification.	Materials and Methods, Lines 82 – 105; Institutional Review Board Statement, Lines 477 – 479.
**Housing and husbandry**	15	Provide details of housing and husbandry conditions, including any environmental enrichment.	Materials and Methods, Lines 84 – 90.
**Animal care and monitoring**	16	a. Describe any interventions or steps taken in the experimental protocols to reduce pain, suffering and distress.	Materials and Methods, Lines 91 – 105, 114 – 116.
		b. Report any expected or unexpected adverse events.	Not explicitly reported.
		c. Describe the humane endpoints established for the study, the signs that were monitored and the frequency of monitoring. If the study did not have humane endpoints, state this.	Materials and Methods, Lines 91 – 99.
**Interpretation/ scientific implications**	17	a. Interpret the results, taking into account the study objectives and hypotheses, current theory and other relevant studies in the literature.	Discussion, Lines 306 – 452.
		b. Comment on the study limitations including potential sources of bias, limitations of the animal model, and imprecision associated with the results.	Not explicitly reported.
**Generalisability/ translation**	18	Comment on whether, and how, the findings of this study are likely to generalise to other species or experimental conditions, including any relevance to human biology (where appropriate).	Discussion, Lines 306 – 452; Conclusions, Lines 454 – 468.
**Protocol registration**	19	Provide a statement indicating whether a protocol (including the research question, key design features, and analysis plan) was prepared before the study, and if and where this protocol was registered.	Not reported.
**Data access**	20	Provide a statement describing if and where study data are available.	Data access,Lines 481-482
**Declaration of interests**	21	a. Declare any potential conflicts of interest, including financial and non-financial. If none exist, this should be stated.	Conflicts of Interest, Line 480.
		b. List all funding sources (including grant identifier) and the role of the funder(s) in the design, analysis and reporting of the study.	Funding, Lines 475 – 476.
