# Supplementary material for: Postauricular gentamicin administration does not provide preferential inner-ear delivery in guinea pigs
Source: PeerJ. 2026 Oct 27;14:e21796. doi: 10.7717/peerj.21796 (PMC13721883; doi:10.7717/peerj.21796)
Supplement: Supplementary file 2 [file peerj-14-21796-s003.pdf]

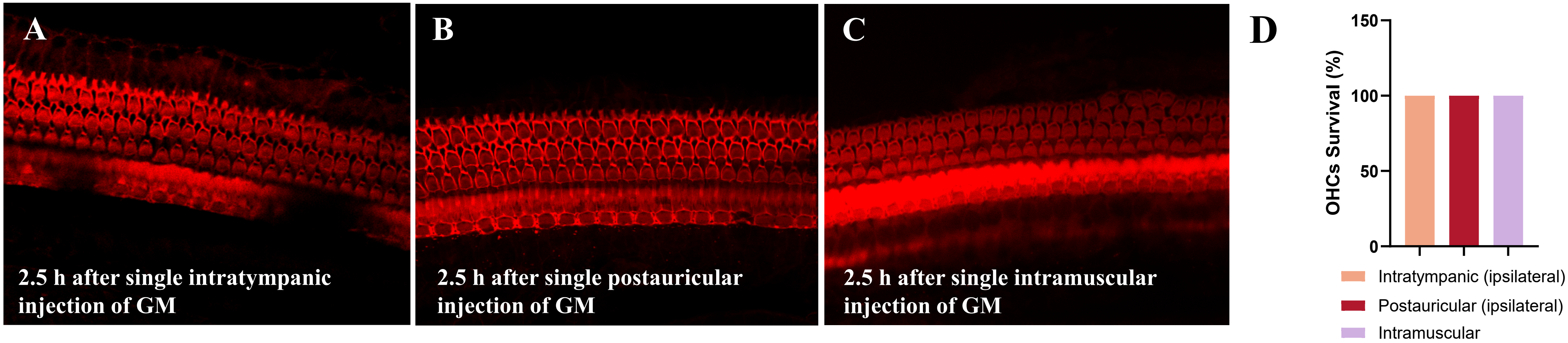
A
2.5 h after single intratympanic injection of GM
B
2.5 h after single postauricular injection of GM
C
2.5 h after single intramuscular injection of GM
D
OHCs Survival (%)
150
100
50
0
Intratympanic (ipsilateral)
Postauricular (ipsilateral)
Intramuscular
