# Supplementary material for: Postauricular gentamicin administration does not provide preferential inner-ear delivery in guinea pigs
Source: PeerJ. 2026 Oct 27;14:e21796. doi: 10.7717/peerj.21796 (PMC13721883; doi:10.7717/peerj.21796)
Supplement: Supplementary file 3 [file peerj-14-21796-s001.zip › Figure 3/Figure 3.pdf]

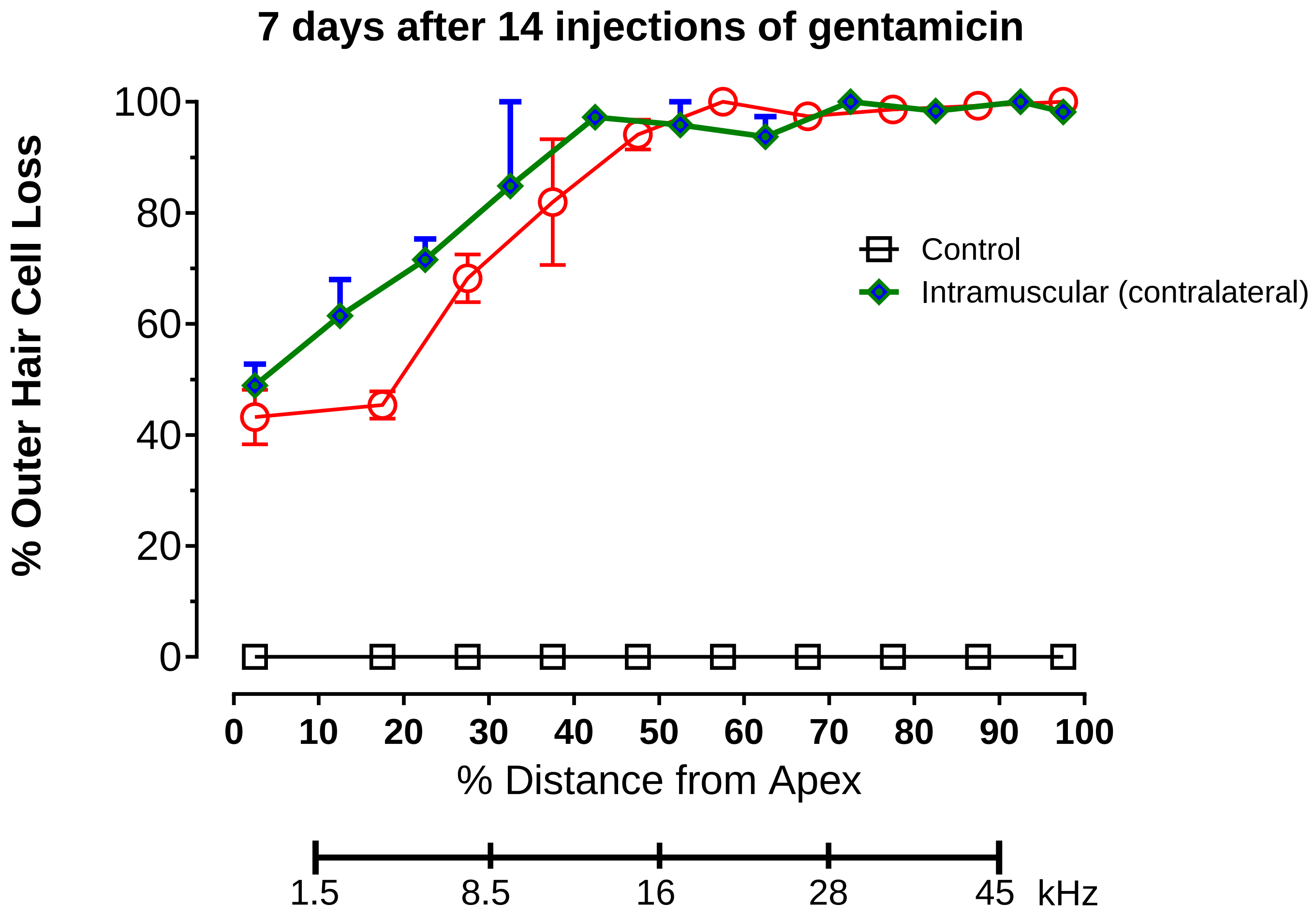

7 days after 14 injections of gentamicin
% Outer Hair Cell Loss
0
20
40
60
80
100
Control
Intramuscular (contralateral)
0
10
20
30
40
50
60
70
80
90
100
% Distance from Apex
1.5
8.5
16
28
45
kHz
