# Supplementary material for: Postauricular gentamicin administration does not provide preferential inner-ear delivery in guinea pigs
Source: PeerJ. 2026 Oct 27;14:e21796. doi: 10.7717/peerj.21796 (PMC13721883; doi:10.7717/peerj.21796)
Supplement: Supplementary file 3 [file peerj-14-21796-s001.zip › Figure 4/Figure 4.pdf]

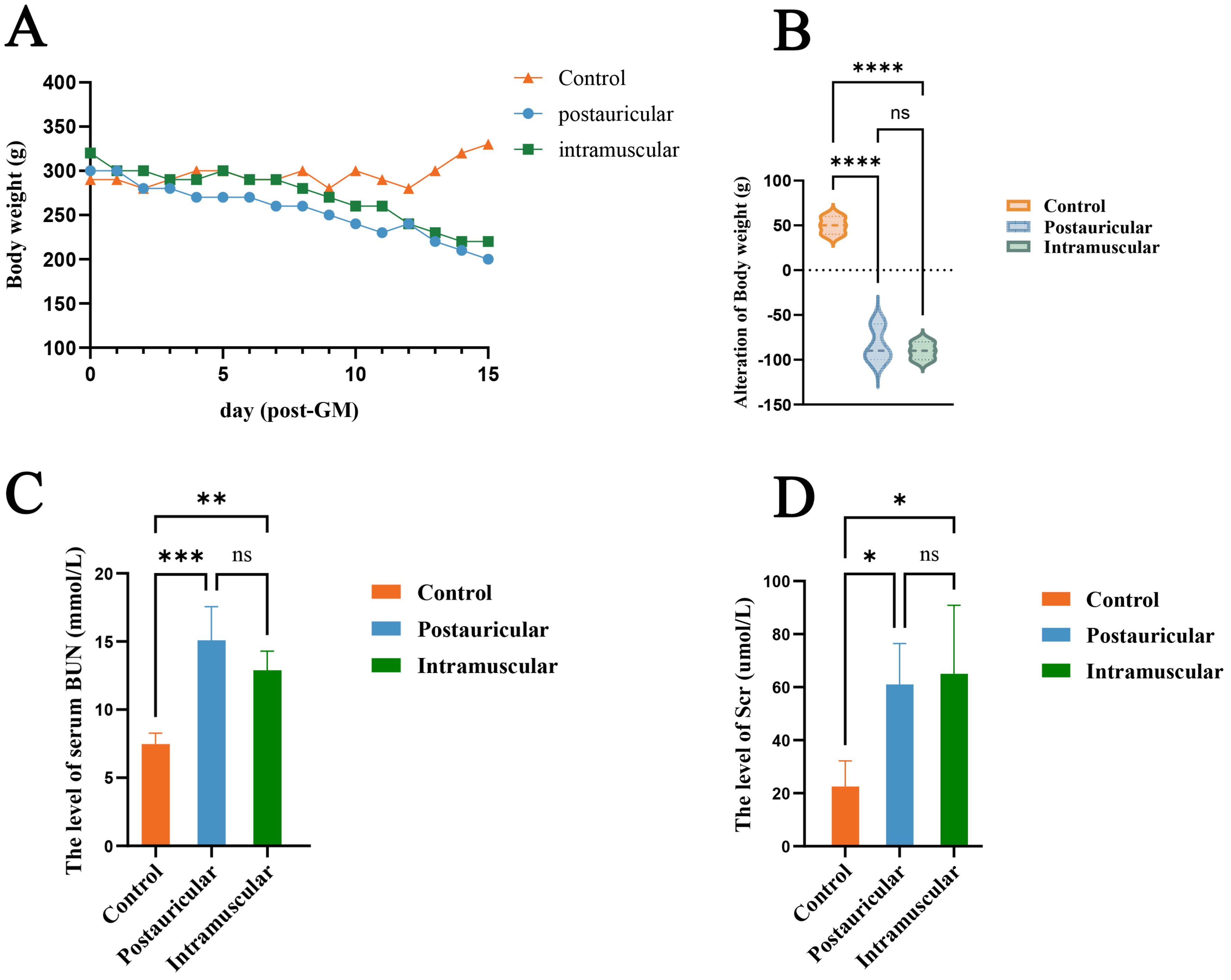
A
Body weight (g)
400
350
300
250
200
150
100
0
5
10
15
day (post-GM)
Control
postauricular
intramuscular
B
****
ns
****
Alteration of Body weight (g)
100
50
0
-50
-100
-150
Control
Postauricular
Intramuscular
C
**
***
ns
The level of serum BUN (mmol/L)
20
15
10
5
0
Control
Postauricular
Intramuscular
Control
Postauricular
Intramuscular
D
*
*
ns
The level of Scr (umol/L)
100
80
60
40
20
0
Control
Postauricular
Intramuscular
Control
Postauricular
Intramuscular
