# Supplementary material for: Postauricular gentamicin administration does not provide preferential inner-ear delivery in guinea pigs
Source: PeerJ. 2026 Oct 27;14:e21796. doi: 10.7717/peerj.21796 (PMC13721883; doi:10.7717/peerj.21796)
Supplement: Supplementary file 3 [file peerj-14-21796-s001.zip › Figure 4/Figure 4A.pdf]

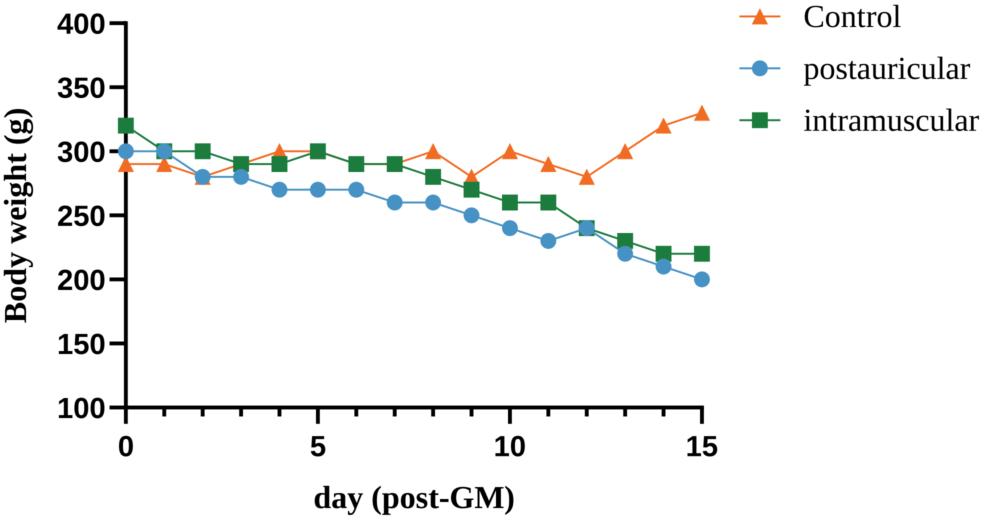

Control
postauricular
intramuscular
400
350
300
250
200
150
100
Body weight (g)
0
5
10
15
day (post-GM)
