# Supplementary material for: Postauricular gentamicin administration does not provide preferential inner-ear delivery in guinea pigs
Source: PeerJ. 2026 Oct 27;14:e21796. doi: 10.7717/peerj.21796 (PMC13721883; doi:10.7717/peerj.21796)
Supplement: Supplementary file 3 [file peerj-14-21796-s001.zip › Figure 4/Figure 4D.pdf]

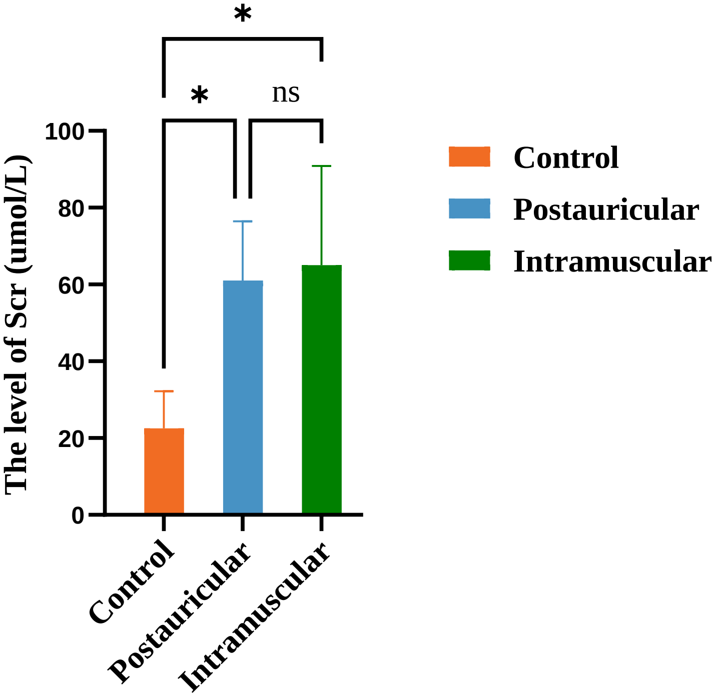
*
*
ns
The level of Scr (umol/L)
100
80
60
40
20
0
Control
Postauricular
Intramuscular
Control
Postauricular
Intramuscular
