# Supplementary material for: Postauricular gentamicin administration does not provide preferential inner-ear delivery in guinea pigs
Source: PeerJ. 2026 Oct 27;14:e21796. doi: 10.7717/peerj.21796 (PMC13721883; doi:10.7717/peerj.21796)
Supplement: Supplementary file 3 [file peerj-14-21796-s001.zip › Figure 6/Figure 6.pdf]

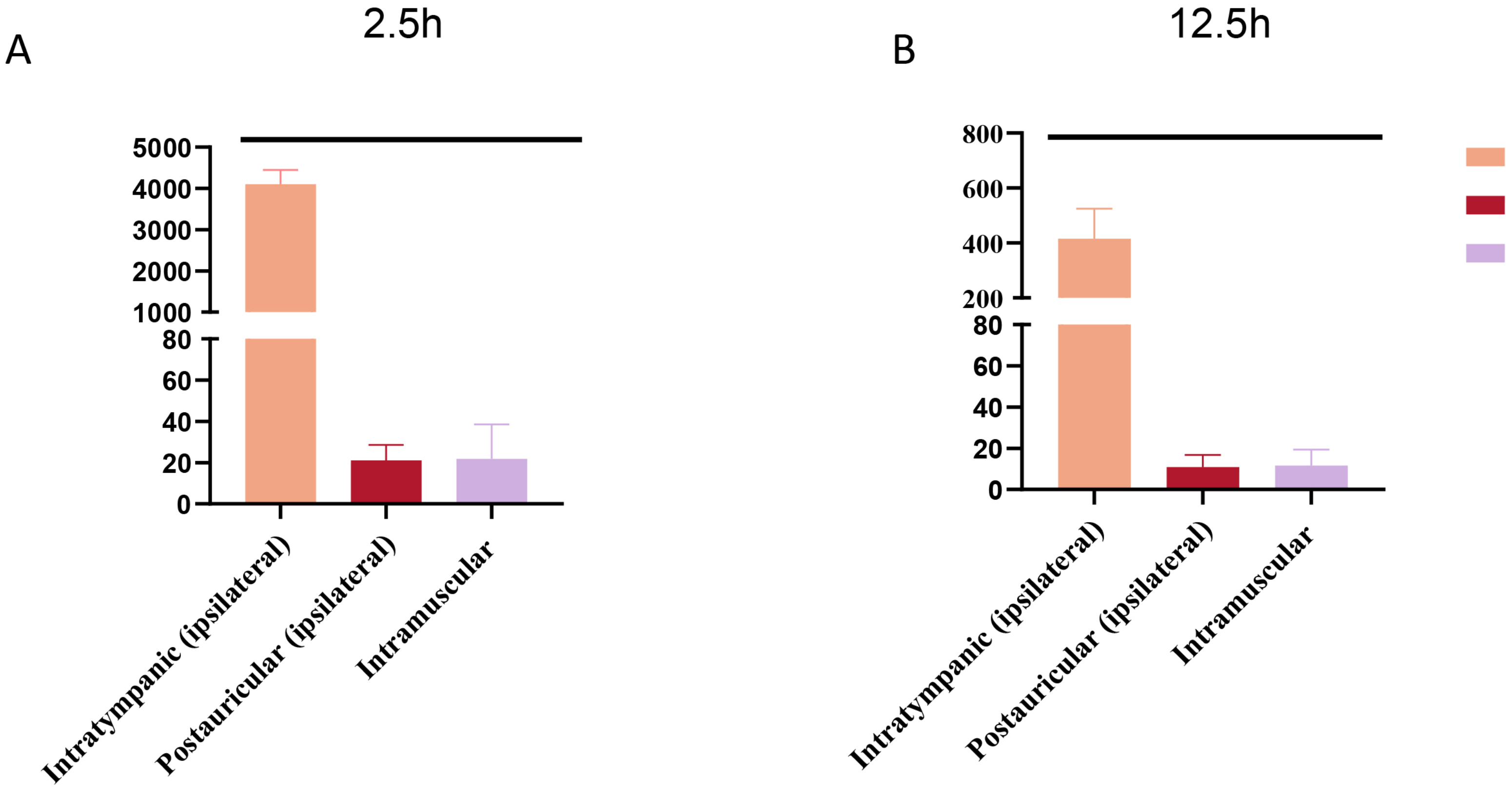

A
2.5h
5000
4000
3000
2000
1000
80
60
40
20
0
Intratympanic (ipsilateral)
Postauricular (ipsilateral)
Intramuscular
B
12.5h
800
600
400
200
80
60
40
20
0
Intratympanic (ipsilateral)
Postauricular (ipsilateral)
Intramuscular
